# Three-dimensional evaluation of palatal bone thickness for insertion of micro implants in a Portuguese population: a retrospective cone-beam CT study

**DOI:** 10.1177/14653125251389975

**Published:** 2025-11-04

**Authors:** Anastasia Ananieva, Iman Bugaighis, Paulo Mascarenhas, Susana Furão, Pedro Mariano Pereira

**Affiliations:** 1 Department of Orthodontics, Egas Moniz School of Health and Science, Campus Universitário, Quinta da Granja Monte de Caparica, Caparica, Almada, Portugal; 2 The Libyan Authority for Scientific Research, Tripoli, Libya; 3 Egas Moniz Center for Interdisciplinary Research (CiiEM); Egas Moniz School of Health & Science, Caparica, Almada, Portugal

**Keywords:** cone-beam computed tomography, palate, orthodontic micro implant, palatal bone thickness

## Abstract

**Objective:**

To examine the palatal bone thickness in a cohort of Portuguese patients representing various age groups to identify optimal insertion sites for enhancing the stability of orthodontic micro implants (MIs). To ascertain whether there are any age- or sex-related variations in palatal bone thickness.

**Design:**

Retrospective observational study.

**Method:**

A total of 50 cone-beam computed tomography (CBCT) scans of patients aged 12–51 years (23 boys/men and 27 girls/women) were analysed. The CBCT scans were grouped according to age and sex. The palatal bone thickness was measured at five points: 4, 8, 12, 16 and 20 mm posterior to the incisive foramen (IF) and at 3, 6 and 9 mm lateral to the midpalatal suture (MPS). A total of 750 regions of interest were evaluated (15 for each of 50 patients). Two-way analysis of variance (ANOVA) and Student’s *t*-tests were employed for data analysis, with a significance level of *P* < 0.05.

**Results:**

The greatest thickness of palatal bone was found to be at 4 mm posterior to the ІF and 9 mm lateral to the MPS in all investigated groups, with a mean thickness of 12.29 ± 2.00 mm for girls/women and 13.59 ± 2.31 mm for boys/men; 13.30 ± 2.38 mm for adolescents and young adults, and 12.27 ± 2.03 mm for adults. Significant statistical differences were identified between male and female individuals and between different age groups (ANOVA, *P* < 0.05).

**Conclusion:**

Palatal bone thickness varied with sex and age; thus, these factors must be considered when selecting the appropriate length of MIs. In general, girls/women had a thinner palatal bone than boys/men. The palatal bone was significantly thicker in the adolescent and young adult age group (12–25 years) than in the adult age group (27–51 years). In addition, bone thickness decreased posteriorly within each sagittal section.

## Plain language summary

Palatal bone thickness for micro implant insertion in Portuguese population

Why was the study done? Micro implants are small screws that can be placed in the intraoral bone during orthodontic treatment to provide support for moving teeth or bone segments. The palate (roof of the mouth) is a favourable region for placing micro implants. But the thickness of the palate may differ between populations. At present, there is no available information on palatal bone thickness in the Portuguese population. This study examined the optimal locations in the palate for positioning micro implants in Portuguese men and women across various age groups. What did the researchers do? A researcher measured the palatal bone thickness on three-dimensional radiographs of 50 Portuguese individuals: 23 men and boys and 27 women and girls aged 12–51 years. Measurements were taken at five points from the middle of the palate (4, 8, 12, 16 and 20 mm) and posterior to the incisive foramen (a small opening behind the front teeth) at 3, 6 and 9 mm lateral to the midline (centre) of the palate. So, 15 measurements were taken from each palate. What did the researchers find? The results showed that bone thickness was different across the palate. But all areas had enough bone thickness to support a micro implant. The thickest bone and best palatal areas were: 1–4 mm behind the incisive foramen and 2–9 mm from the midline of the palate. Men had thicker palatal bones than women, and younger patients had thicker palatal bones than older ones. What do the findings mean? These findings help orthodontists choose the best locations in the palate for micro implant placement in Portuguese patients.

## Introduction

Micro implants (MIs) have been increasingly utilised as an effective additional anchorage modality in orthodontic practice. MI versatile biomechanics, minimal invasiveness, cost-effectiveness and relative ease of insertion contributed to their acceptance by orthodontists and patients, expanding orthodontic treatment boundaries (Chang et al., 2021; Lee et al., 2021).

The most common MI insertion site is the vestibular inter-radicular alveolar process. A significant limitation of this location is its variable bone density (Chugh et al., 2013), proximity to dental roots and soft-tissue properties, leading to a failure rate approaching 13.5% (Alharbi et al., 2018). In 1996, Wehrbein et al. (1996) first reported the possibility of inserting MIs in the anterior palate. The subsequent clinical recommendation suggested that ideal MI palatal insertion sites are located at 4–20 mm posterior to the incisive foramen and 3–9 mm lateral to the midpalatal suture (MPS), an area referred to as the T-zone (Holm et al., 2016; Іodice et al., 2022; Ludwig et al., 2011; Wilmes et al., 2016). Wilmes et al. (2016) determined the T-zone to be an ideal location for the insertion of MІs, highlighting the advantages of this region, such as high bone density, the relatively thin keratinised soft tissue overlying the T-zone making the insertion procedure easier and reducing complication risks. In addition, this anatomical region just posterior to the third palatal rugae is located away from critical structures like tooth roots, minimising the risk of damage during insertion. The T-zone allows for both median and paramedian MI insertion, thereby providing flexibility in treatment planning contingent upon patient-specific orthodontic needs, which is conducted away from vital structures (Holm et al., 2016; Іodice et al., 2022; Ludwig et al., 2011).

Recently, numerous orthodontic devices have been integrated with palatal MI, such as maxillary expansion devices with skeletal anchorage in adolescents and young adults (Carlson et al., 2016; MacGinnis et al., 2014), and molar mesialisation and distalisation appliances (Ludwig et al., 2011). Biomechanical consideration might require inserting MIs in other sites in the posterior palate to attain a planned orthodontic tooth movement. Bone thickness is critical for stabilising the infra bony screw segment of the MI (Hourfar et al., 2015; Ludwig et al., 2011; Wilmes et al., 2016). It is, therefore, crucial to ascertain the total and cortical bone thickness of the entire palate, given that the stability of the MI is primarily achieved through mechanical retention rather than osteointegration (Bourassa et al., 2018; Migliorati et al., 2012). Otherwise, the primary stability of the MI might be compromised (Ichinohe et al., 2019). MI length needs to have the same or less length than the depth of the palate, otherwise, the tip of the MI would penetrate the nasal/sinus floor. There is uncertainty among numerous orthodontists regarding the side effects of this penetration. Several clinicians reported the development of irritation and other adverse consequences in the nose and sinuses (Truong et al., 2022). Others favour this penetration to achieve a bicortical anchorage to enhance MI stability further, debating that there are rare, published incidents of nasal or maxillary sinuses deleterious effects after MI use (Kravitz and Kusnoto, 2007).

Previous studies measuring palatal bone thickness have employed cone-beam computed tomography (CBCT) (Chang et al., 2021; Vidalon et al., 2021; Wilmes et al., 2016). CBCT has the advantage of enabling the generation of a three-dimensional (3D) representation of the selected site. Subsequently, the image area’s slice can be viewed from any angle and digitally saved (Bonangi et al., 2018; Nakahara et al., 2012).

Numerous studies have yielded significant insights into palatal bone thickness and its impact on orthodontic MI placement. Notably, Nishi et al. (2014) reported significant differences in the palatal bone thickness among the four ethnic groups they examined. These investigators, therefore, recommended examining the palatal thickness for each population to provide a comprehensive understanding of variations in palatal bone thickness and their implications for MI placement. To date, no similar study has been conducted on the Portuguese population. Therefore, this study aimed to investigate palatal bone thickness in a cohort of Portuguese patients from different age groups to identify favourable insertion sites for optimal stability of orthodontic MIs and to determine potential age- or sex-related variations in palatal bone thickness. The null hypotheses tested in this investigation were as follows:

1. There is no significant difference in the mean palatal bone thickness across different measurement points on the palate.
2. There is no significant difference in the mean palatal bone thickness between male and female individuals at the specific measurement points.
3. There is no significant difference in the mean palatal bone thickness between adolescents and young adults, and adults at the specific measurement points.

## Materials and methods

This retrospective quantitative observational investigation screened 614 CBCT scans performed for diagnostic purposes on dental patients of various age groups and sexes who had visited the Egas Moniz University Clinics between January 2023 and March 2024. The research protocol was reviewed and approved by the Ethics Committee of the Egas Moniz School of Health and Science (PT-201/23). Before conducting the CBCT examinations, informed consent was obtained from all patients. Patients with confounding factors that might affect palatal thickness were excluded from the study. These included cleft lip and palate, craniofacial deformities, systemic diseases, the use of medications that might affect bone density, hypodontia or supernumerary teeth, impacted teeth or cysts, and prior surgical procedures, including extractions or extensive metal restorations that might affect the quality of the CBCT. This investigation was performed exclusively on Portuguese patients with fully erupted maxillary permanent dentition, including the second molars. It was reported according to the Strengthening the Reporting of Observational Studies in Epidemiology (STROBE) guidelines for observational studies (Bruggesser et al., 2023).

### Sample size

**Figure 1.**
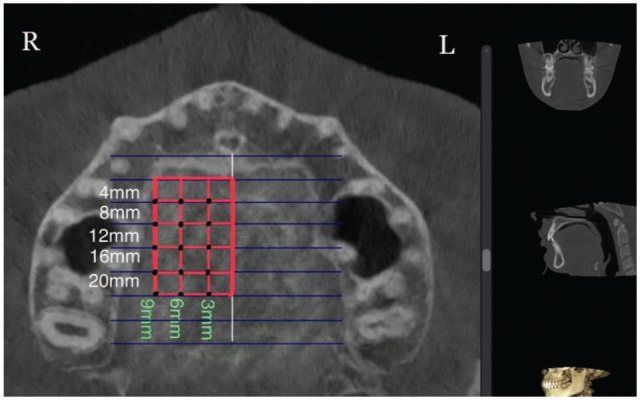
All assigned points located at 4, 8, 12, 16 and 20 mm posterior to the incisive foramen and 3, 6 and 9 mm lateral to the midpalatine suture (right side, axial view).

A convenience sample of 50 CBCT scans fulfilled the inclusion criteria of the present investigation. Most analyses recommended a minimum sample size of 30 (power = 80%, alpha = 0.05). The achieved power with 50 scans is 99.9%. This number reflected the retrospective nature of the study and the ethical limitation on unnecessary radiation exposure (Figure 1). The observed effect size (Cohen’s *d* = 3.77) yielded a statistical power >99.9% (α = 0.05, two-tailed). Moreover, the associated *P* value (*P* < 10^–³³^) remained highly significant after the Benjamini–Hochberg (BH) correction for false discovery rate (FDR), indicating that the study was adequately powered to detect relevant anatomical differences even under multiple testing controls.

### Image acquisition and processing

All CBCT scans were obtained using Planmeca Viso G7 (Planmeca, Helsinki, Finland). The following settings were used: 120 kVp, 5 mA, large field of view (20 cm × 17 cm), exposure time of 30 s and slice thickness of 0.45 mm. The 3D images were constructed using Romexis Viewer version 6.0 (Planmeca, Helsinki, Finland).

### Standardisation and landmarks identification

The personal details of each patient were inserted into an Excel file. A coded number was assigned to each included CBCT scan to blind the examiner from possible bias concerning patient age and sex. The CBCT analyses were performed by an orthodontist who had undergone training sessions using the software Romexis Viewer version 6.0 (Planmeca, Helsinki, Finland).

**Figure 2.**
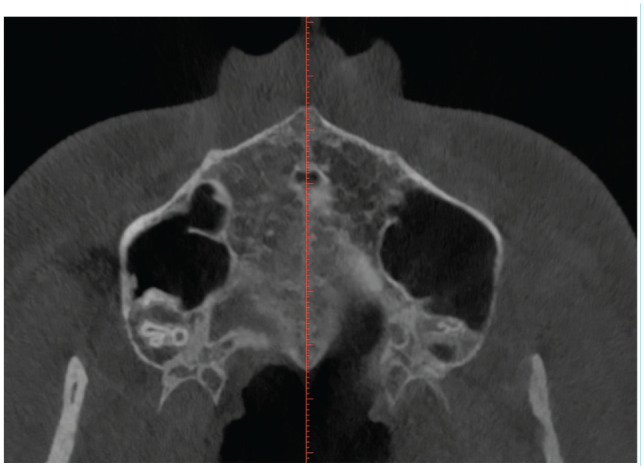
Axial view of the incisive foramen and midpalatine suture.

**Figure 3.**
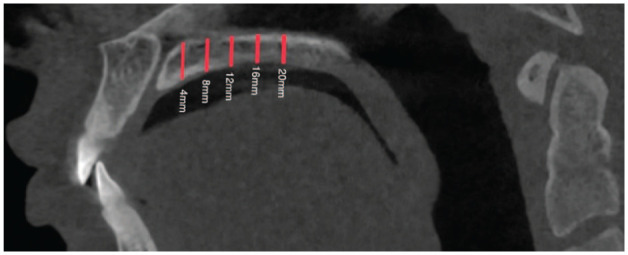
Sagittal view of the incisive foramen and midpalatine suture in relation to the measurement points located at 4, 8, 12, 16 and 20 mm posterior to the incisive foramen.

**Figure 4.**
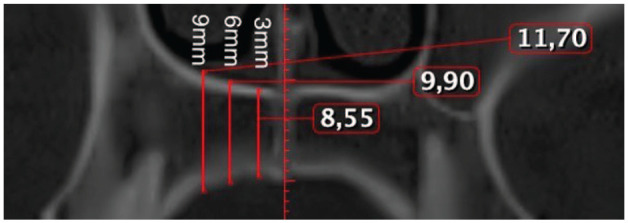
All assigned points located at 4, 8, 12, 16 and 20 mm posterior to the incisive foramen and 3, 6 and 9 mm lateral to midpalatine suture (right side, axial view).

Before analysis, each image was aligned to a standard position. The distal margin of the incisive foramen (ІF) and the posterior nasal spine (PNS) were identified as anatomical reference landmarks on the axial view (Figure 2), and a reference line was drawn across the incisive foramen in the midline on the same view. This plane was then tracked in the sagittal (Figure 3) and axial view (Figure 2). The palatal bone thickness was assessed on the coronal plane (Figure 4), where the measurement points had to be defined in the coronal and sagittal planes over a set of evenly sized grids over the areas of interest.

In the sagittal plane, measurement points were taken in the anterior region of the palate at 4 and 8 mm and in the posterior region at 12, 16 and 20 mm distally from the IF (Figure 1). These points were designated as S4, S8, S12, S16 and S20, respectively. Vertical lines were drawn perpendicular to the reference line through these points. Furthermore, commencing at point S4 (and continuing to S8, S12, S16 and S20) and transitioning to the coronal view (Figure 4), the measurements were taken exclusively on the right side. The lateral aspect of each patient was measured at 3, 6 and 9 mm from the midline (designated as C3, C6 and C9, respectively) (Figure 4). Prior studies have indicated no significant differences in palatal bone thickness between the right and left sides of the palate (Gracco et al., 2008).

**Figure 5.**
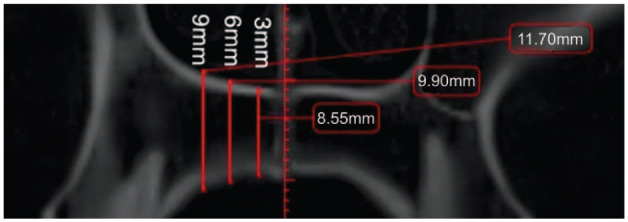
All assigned points located at 4, 8, 12, 16 and 20 mm posterior to the incisive foramen and 3, 6 and 9 mm lateral to midpalatine suture (right side, axial view).

A total of 750 measurements were recorded, with 15 measurements taken for each of the 50 patients. In addition, the points situated 4 and 8 mm posterior to the IF and 3, 6 and 9 mm lateral to the MPS were designated the ‘anterior region’. The points located 12, 16 and 20 mm posterior to the IF and 3, 6 and 9 mm lateral to the MPS were consequently designated as the ‘posterior region’ (Figure 5). Each measurement was subsequently entered into an Excel worksheet for further statistical data analysis.

### Statistical analysis

Statistical Package for the Social Sciences version 28 (IBM Corp., Armonk, NY, USA) was utilised for the statistical analysis. Descriptive statistics included the mean, standard deviation (SD) and 95% confidence interval. To assess significant differences in measurements between age and sex groups, the Student’s *t*-test was applied in the results tables. The normality of the data distribution was evaluated using the Shapiro–Wilk test, while Levene’s test assessed the homogeneity of variance in bone thickness across different points and groups. When minor deviations from normality were detected, the Student’s *t*-test results were further verified using a non-parametric equivalent, the Mann–Whitney U-test. To control for the FDR, the BH procedure was implemented. Moreover, Box’s test was employed to evaluate the assumption of equality of covariance matrices across the groups defined by age and sex. The objective of the test was to ascertain whether the observed covariance matrices of the dependent variables are equal across groups, a critical assumption for the validity of analysis of variance (ANOVA). A between-patient ANOVA was conducted to evaluate the effect of age and sex on the average bone thickness. The analysis was conducted using the type III sum of squares method, with a significance level of 0.05.

A mixed-effects regression analysis was employed to evaluate the effects of sex (factor), age (continuous covariate) and point (indicator of the 15 measurements), taking into account the correlation of repeated measurements. Subsequently, a hierarchical cluster analysis was performed to screen for homogeneous points. The homogeneity results from cluster analysis provide a clinically meaningful way to predict suitable areas for MI insertion. Identifying regions with stable and uniform bone thickness can enhance treatment predictability, while recognising variable zones ensures proper individualised planning to avoid complications.

### Reproducibility study

The examiner conducted a re-evaluation of 10 CBCT scans, selected at random, which had previously undergone measurement of the same 150 points of interest at 2-week intervals. The intraclass correlation (ICC) for agreement and Bland and Altman’s plot analysis were employed to assess the degree of similarity between the measurements taken at two different times. Two forms of ICC were computed: ICC for single measurements, which reflects the reliability of individual measurements taken at different time points, and ICC for average measurements, which assesses the reliability when multiple measurements are averaged, thereby reducing variability and increasing precision.

## Results

A total of 50 participants met the established inclusion criteria, comprising 23 boys/men and 27 girls/women aged 12–51 years. The sample was further divided into two age groups to facilitate a more nuanced analysis: adolescents and young adults (13 male and 12 female adolescents aged 12–25 years) and adults (10 men and 15 women aged 27–51 years).

**Figure 6.**
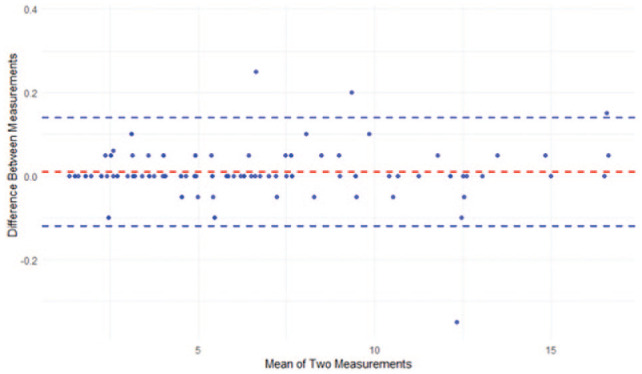
Bland–Altman plot illustrating the agreement between two repeated measurements. The mean difference (bias) is shown as a dashed red line, while the blue dashed lines represent the 95% limits of agreement (LOA).

**Figure 7.**
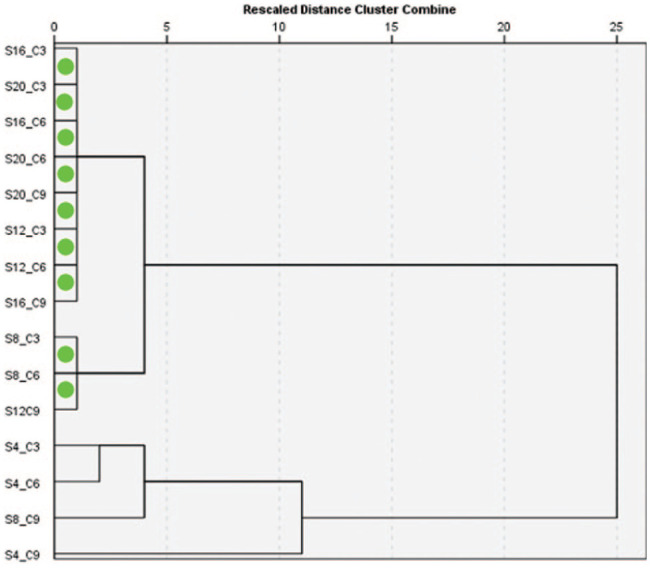
Dendrogram of linkage between groups according to thickness. The green circles show the two groups of points that are most similar.

**Figure 8.**
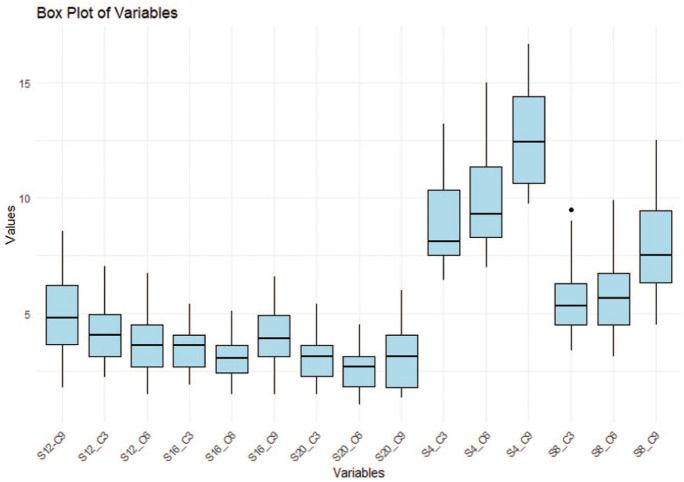
Box plot of average palatal bone thickness point. The boxes represent the IQR, with the median shown as a bold horizontal line. Whiskers extend to the minimum and maximum values within 1.5 times the IQR, while outliers are shown as individual points. IQR, interquartile range.

The findings of the reliability analyses suggest that the single measurements possess high reliability (ICC = 0.50, 95% CI = 0.40–0.61), yet the mean measurements exhibit even higher reliability (ICC = 0.94, 95% CI = 0.91–0.96). In addition, the ICC assessment demonstrated consistently high agreement between repeated measures, with values in the range of approximately 0.99–1. This indicates an extremely strong concordance both at individual measurement points and across patients, suggesting that patient variability far exceeds measurement discrepancy (Table 1). Furthermore, the Bland–Altman plot analysis (Figure 6) reveals a low bias, thereby suggesting a high level of agreement between the two repeated measurements. This finding also supports the hypothesis of good intra-examiner reliability, indicating that the examiner is consistent in their measurements. The absence of significant heteroscedasticity (an increase in variability with larger values) is observed among the variables (Figure 7). The Box’s test results indicated no statistically significant difference (*P* = 0.515) between the covariance matrices of the groups (Figure 8), suggesting that the patterns of point thickness change similarly across the different age and sex groups investigated (Table 2).

**Table 1.** Displays the results of the ICC analysis and the 95% CI of the reproducibility study.

	ICC	95% CI
Unique measurements	0.497	0.396–0.614
Average measurements	0.937	0.908–0.960

CI, confidence interval; ICC, intraclass correlation coefficient.

**Table 2.** Box’s test: tests the null hypothesis that the observed covariance matrices of the dependent variable (‘points’) are equal across groups.

Test*	Statistic	df1	df2	F	*P*
Box’s test of equality of covariance matrices	180.6	120	6761	1.0	0.515
*Tests of between-patient effects*					
Intercept	3145.5	1	–	201.7	<0.001
Age	43.0	1	47	2.8	0.103
Sex	84.9	1	47	5.5	0.024
Error	733.0	47	–	–	–

The non-significant *P* value (0.515) suggests that the assumption of homogeneity of covariance is met. Between-patient effects: The variable ‘sex’ had a significant effect on the dependent variable (*P* = 0.024), whereas ‘age’ did not (*P* = 0.103).

* The model included Intercept + Age + Sex as factors.

The Shapiro–Wilk and Levene’s tests revealed that most of the group distributions were found to be normal, exhibiting comparable variances among their respective groups. However, there were exceptions, which included minor violations. For these exceptions, the Mann–Whitney test results exhibited a concordant level of pairwise significance with those of Student’s *t*-test.

The two-way ANOVA test for sex and age group revealed significant between-patient effects (*P* < 0.001), indicating overall discrepancies in the group’s mean measurements. Subsequent analyses demonstrated that sex had a significant effect on the measurements (*P* = 0.024), whereas age group did not (*P* = 0.103) (Table 2).

Figure 6 demonstrates that across all the samples analysed, the greatest palatal bone thickness was in the anterior region of the palate (4–8 mm distally to the ІF). This thickness decreased in a posterior direction within each sagittal section. The maximum palatal bone thickness was observed at a point 4 mm posterior to the ІF and 9 mm lateral to the MPS, specifically at the S4/C9 coordinates. The mean thickness at this point was 12.29 ± 2.00 mm for girls/women and 13.59 ± 2.31 mm for boys/men. Notably, other points with high bone thickness values were identified, including S4/C3 (8.32 ± 1.46 mm for girls/women, 9.77 ± 1.91 mm for boys/men), S4/C6 (9.19 ± 1.66 mm for girls/women, 10.75 ± 2.46 mm for boys/men) and S8/C9 (7.12 ± 1.89 mm for girls/women, 8.63 ± 1.95 mm for boys/men), as detailed in Table 3.

**Table 3.** Differences between pairs of sites (in mm).

	S4/C3	S4/C6	S4/C9	S8/C3	S8/C6	S8/C9	S12/C3	S12/C6	S12/C9	S16/C3	S16/C6	S16/C9	S20/C3	S20/C6	S20/C9
S4/ C3	–	−0.92 (−1.30–−0.55)*	−3.90 (−4.46–−3.33)*	3.38 (3.08–3.68)*	3.11 (2.72–3.50)*	1.18 (0.58–1.77)*	4.92 (4.50–5.34)*	5.34 (4.86–5.83)*	4.09 (3.60–4.57)*	5.55 (5.04–6.05)*	5.96 (5.44–6.47)*	5.10 (4.60–5.60)*	5.96 (5.41–6.51)*	6.39 (5.88–6.91)*	5.82 (5.31–6.33)*
S4/ C6	0.92 (0.55–1.30)*	–	−2.97 (−3.32–−2.63)*	4.30 (3.88–4.73)*	4.03 (3.68–4.38)*	2.10 (1.65–2.55)*	5.84 (5.33–6.35)*	6.26 (5.75–6.78)*	5.01 (4.54–5.48)*	6.47 (5.86–7.08)*	6.88 (6.31–7.45)*	6.02 (5.51–6.53)*	6.88 (6.23–7.53)*	7.32 (6.72–7.91)*	6.74 (6.19–7.30)*
S4/ C9	3.90 (3.33–4.46)*	2.97 (2.63–3.32)*	–	7.28 (6.72–7.83)*	7.00 (6.56–7.45)*	5.07 (4.64–5.50)*	8.82 (8.21–9.42)*	9.24 (8.66–9.82)*	7.99 (7.48–8.49)*	9.44 (8.77–10.12)*	9.85 (9.25–10.45)*	9.00 (8.46–9.54)*	9.86 (9.18–10.53)*	10.29 (9.68–10.90)*	9.72 (9.17–10.27)*
S8/ C3	−3.38 (−3.68–−3.08)*	−4.30 (−4.73–−3.88)*	−7.28 (−7.83–−6.72)*	–	−0.27 (−0.56–0.01)	−2.21 (−2.76–−1.65)*	1.54 (1.27–1.81)*	1.96 (1.65–2.28)*	0.71 (0.33–1.09)*	2.17 (1.79–2.55)*	2.58 (2.18–2.97)*	1.72 (1.31–2.13)*	2.58 (2.15–3.01)*	3.01 (2.61–3.41)*	2.44 (2.03–2.85)*
S8/ C6	−3.11 (−3.50–−2.72)*	−4.03 (−4.38–−3.68)*	−7.00 (−7.45–−6.56)*	0.27 (−0.01–0.56)	–	−1.93 (−2.30–−1.57)*	1.81 (1.46–2.17)*	2.24 (1.93–2.55)*	0.98 (0.68–1.29)*	2.44 (2.01–2.87)*	2.85 (2.48–3.22)*	1.99 (1.63–2.36)*	2.85 (2.39–3.32)*	3.29 (2.88–3.69)*	2.71 (2.32–3.11)*
S8/ C9	−1.18 (−1.77–−0.58)*	−2.10 (−2.55–−1.65)*	−5.07 (−5.50–−4.64)*	2.21 (1.65–2.76)*	1.93 (1.57–2.30)*	–	3.75 (3.22–4.27)*	4.17 (3.69–4.64)*	2.91 (2.49–3.33)*	4.37 (3.77–4.97)*	4.78 (4.27–5.29)*	3.93 (3.43–4.42)*	4.79 (4.18–5.39)*	5.22 (4.66–5.77)*	4.64 (4.12–5.17)*
S12/C3	−4.92 (−5.34–−4.50)*	−5.84 (−6.35–−5.33)*	−8.82 (−9.42–−8.21)*	−1.54 (−1.81–−1.27)*	−1.81 (−2.17–−1.46)*	−3.75 (−4.27–−3.22)*	–	0.42 (0.19–0.65)*	−0.83 (−1.19–−0.48)*	0.63 (0.42–0.84)*	1.04 (0.79–1.28)*	0.18 (−0.19–0.55)	1.04 (0.78–1.31)*	1.47 (1.20–1.75)*	0.90 (0.54–1.26)*
S12/C6	−5.34 (−5.83–−4.86)*	−6.26 (−6.78–−5.75)*	−9.24 (−9.82–−8.66)*	−1.96 (−2.28–−1.65)*	−2.24 (−2.55–−1.93)*	−4.17 (−4.64–−3.69)*	−0.42 (−0.65–−0.19)*	–	−1.25 (−1.50–−1.01)*	0.20 (−0.07–0.48)	0.61 (0.42–0.81)*	−0.24 (−0.52–0.03)	0.62 (0.32–0.92)*	1.05 (0.80–1.30)*	0.48 (0.19–0.76)*
S12/C9	−4.09 (−4.57–−3.60)*	−5.01 (−5.48–−4.54)*	−7.99 (−8.49–−7.48)*	−0.71 (−1.09–−0.33)*	−0.98 (−1.29–−0.68)*	−2.91 (−3.33–−2.49)*	0.83 (0.48–1.19)*	1.25 (1.01–1.50)*	–	1.46 (1.04–1.88)*	1.87 (1.57–2.16)*	1.01 (0.76–1.27)*	1.87 (1.44–2.30)*	2.31 (1.95–2.66)*	1.73 (1.41–2.05)*
S16/C3	−5.55 (−6.05–−5.04)*	−6.47 (−7.08–−5.86)*	−9.44 (−10.12–−8.77)*	−2.17 (−2.55–−1.79)*	−2.44 (−2.87–−2.01)*	−4.37 (−4.97–−3.77)*	−0.63 (−0.84–−0.42)*	−0.20 (−0.48–0.07)	−1.46 (−1.88–−1.04)*	–	0.41 (0.18–0.64)*	−0.45 (−0.84–−0.05)	0.41 (0.25–0.57)*	0.85 (0.60–1.09)*	0.27 (−0.10–0.64)
S16/C6	−5.96 (−6.47–−5.44)*	−6.88 (−7.45–−6.31)*	−9.85 (−10.45–−9.25)*	−2.58 (−2.97–−2.18)*	−2.85 (−3.22–−2.48)*	−4.78 (−5.29–−4.27)*	−1.04 (−1.28–−0.79)*	−0.61 (−0.81–−0.42)*	−1.87 (−2.16–−1.57)*	−0.41 (−0.64–−0.18)*	–	−0.86 (−1.10–−0.61)*	0.00 (−0.23–0.24)	0.44 (0.24–0.63)*	−0.14 (−0.39–0.12)
S16/C9	−5.10 (−5.60–−4.60)*	−6.02 (−6.53–−5.51)*	−9.00 (−9.54–−8.46)*	−1.72 (−2.13–−1.31)*	−1.99 (−2.36–−1.63)*	−3.93 (−4.42–−3.43)*	−0.18 (−0.55–0.19)	0.24 (−0.03–0.52)	−1.01 (−1.27–−0.76)*	0.45 (0.05–0.84)	0.86 (0.61–1.10)*	–	0.86 (0.48–1.24)*	1.29 (1.01–1.57)*	0.72 (0.53–0.91)*
S20/C3	−5.96 (−6.51–−5.41)*	−6.88 (−7.53–−6.23)*	−9.86 (−10.53–−9.18)*	−2.58 (−3.01–−2.15)*	−2.85 (−3.32–−2.39)*	−4.79 (−5.39–−4.18)*	−1.04 (−1.31–−0.78)*	−0.62 (−0.92–−0.32)*	−1.87 (−2.30–−1.44)*	−0.41 (−0.57–−0.25)*	−0.00 (−0.24–0.23)	−0.86 (−1.24–−0.48)*	–	0.43 (0.22–0.64)*	−0.14 (−0.50–0.22)
S20/C6	−6.39 (−6.91–−5.88)*	−7.32 (−7.91–−6.72)*	−10.29 (−10.90–−9.68)*	−3.01 (−3.41–−2.61)*	−3.29 (−3.69–−2.88)*	−5.22 (−5.77–−4.66)*	−1.47 (−1.75–−1.20)*	−1.05 (−1.30–−0.80)*	−2.31 (−2.66–−1.95)*	−0.85 (−1.09–−0.60)*	−0.44 (−0.63–−0.24)*	−1.29 (−1.57–−1.01)*	−0.43 (−0.64–−0.22)*	–	−0.57 (−0.79–−0.35)*
S20/C9	−5.82 (−6.33–−5.31)*	−6.74 (−7.30–−6.19)*	−9.72 (−10.27–−9.17)*	−2.44 (−2.85–−2.03)*	−2.71 (−3.11–−2.32)*	−4.64 (−5.17–−4.12)*	−0.90 (−1.26–−0.54)*	−0.48 (−0.76–−0.19)*	−1.73 (−2.05–−1.41)*	−0.27 (−0.64–0.10)	0.14 (−0.12–0.39)	−0.72 (−0.91–−0.53)*	0.14 (−0.22–0.50)	0.57 (0.35–0.79)*	–

Values are given as mean (95% confidence interval).

Table 4 further illustrates that boys/men exhibited significantly greater average palatal bone thickness than girls/women (*P* < 0.05) at all points located 4 and 12 mm posterior to the IF, and at one point located 8 mm posterior to the IF. Notably, no significant differences in thickness were observed between male and female individuals at the points situated 16 and 20 mm posterior to the IF.

**Table 4.** Descriptive statistics of palatal bone thickness according to sex (in mm), determined by *t*-test (*P* ⩽ 0.05).

Points	Female (n = 27)	Male (n = 23)	Adjusted *P* value
S4/C3	8.32 ± 1.46	9.77 ± 1.91	**0.043**
S4/C6	9.19 ± 1.66	10.75 ± 2.46	**0.043**
S4/C9	12.29 ± 2.00	13.59 ± 2.31	**0.041**
S8/C3	5.16 ± 1.14	6.13 ± 1.69	0.053
S8/C6	5.50 ± 1.25	6.33 ± 1.88	0.133
S8/C9	7.12 ± 1.89	8.63 ± 1.95	**0.043**
S12/C3	3.75 ± 1.00	4.44 ± 1.01	**0.043**
**S12/C6**	3.22 ± 0.96	4.14 ± 1.32	**0.042**
**S12/C9**	4.41 ± 1.48	5.47 ± 1.52	**0.040**
**S16/C3**	3.38 ± 1.02	3.52 ± 0.96	0.727
**S16/C6**	2.87 ± 0.94	3.23 ± 0.91	0.267
**S16/C9**	3.73 ± 1.51	4.07 ± 1.23	0.523
**S20/C3**	3.06 ± 1.03	2.99 ± 0.85	0.790
**S20/C6**	2.63 ± 0.71	2.56 ± 0.91	0.790
**S20/C9**	3.05 ± 1.22	3.31 ± 1.21	0.561

Values are given as mean ± SD. Red: anterior region.

The significant P values are written in Bold (*p* < 0.5). IF, incisive foramen; MPS, midpalatal suture.

Similar to what was observed in the sex groups, the age groups exhibited the highest value at the S4/C9 point, with a mean thickness of 13.30 ± 2.38 mm for adolescents and young adults and 12.27 ± 2.03 mm for adults (Table 5). In addition, thicker bone was noticed at the following points: S4/C3, S4/C6, S8/C3, S8/C6 and S8/C9 (Table 4). On average, adolescents and young adults exhibited higher mean palatal bone thickness than adults. Significant statistical differences were identified at 8, 12 and 16 mm posterior to the IF (*P* < 0.05).

**Table 5.** Descriptive statistics of palatal bone thickness according to age (in mm).

Points	Adolescents/young adults (13 M, 12 F; age range = 12–25 years)	Adults (10 M, 15 F; age range = 27–51 years)	Adjusted *P* value
S4/C3	9.08 ± 1.69	8.9 ± 1.97	0.772
S4/C6	10.32 ± 2.10	9.51 ± 2.24	0.223
S4/C9	13.30 ± 2.38	12.27 ± 2.03	0.223
S8/C3	5.57 ± 1.37	5.64 ± 1.63	0.870
S8/C6	6.38 ± 1.57	5.38 ± 1.52	0.066
S8/C9	8.75 ± 1.92	6.88 ± 1.74	**0.006**
**S12/C3**	4.27 ± 0.97	3.87 ± 1.12	0.223
**S12/C6**	4.05 ± 1.17	3.25 ± 1.15	0.056
**S12/C9**	5.57 ± 1.36	4.21 ± 1.49	**0.006**
**S16/C3**	3.63 ± 0.97	3.25 ± 0.98	0.223
**S16/C6**	3.49 ± 0.83	2.58 ± 0.81	**0.004**
**S16/C9**	4.40 ± 0.10	3.38 ± 1.47	**0.031**
**S20/C3**	3.24 ± 0.92	2.81 ± 0.94	0.222
**S20/C6**	2.76 ± 0.89	2.43 ± 0.68	0.223
**S20/C9**	3.41 ± 1.13	2.93 ± 1.26	0.223

Values are given as mean ± SD.

The significant P values are written in Bold (*p* < 0.5). F, female; IF, incisive foramen; M, male; MPS, midpalatal suture.

The cluster analysis illustrated in Figure 7 indicates a high degree of homogeneity. The first homogeneous group is formed by the points S16/C3, S20/C3, S16/C6, S20/C9, S12/C3, S12/C6 and S16/C9. These measurements originate from the posterior region of the hard palate, situated between 12 and 20 mm posterior to the IF. The second homogeneous group is formed by the S8/C3, S8/C6 and S12/C9 points, predominantly located in the anterior part of the palate.

Table 6 presents a summary of the mixed-effects model, which suggests that, on average, boys/men had a mean of 0.68 mm greater bone thickness compared to girls/women (*P* = 0.024). Conversely, there was a 0.03-mm decrease in bone thickness for each additional year of age; however, this effect was not statistically significant (*P* = 0.103).

**Table 6.** Summary of the mixed-effects model results.

Effect	Estimate	Standard error	DF	t value	*P* value
(Intercept)	9.367904	0.4995971	686	18.750918	<0.0001
Sex	0.682007	0.2922327	47	2.333782	0.0239
Age	−0.025953	0.0156243	47	−1.661068	0.1034
S4/C3	0.922800	0.2132179	686	4.327967	<0.0001
S4/C6	3.897800	0.2132179	686	18.280829	<0.0001
S4/C9	−3.380200	0.2132179	686	−15.853266	<0.0001
S8/C3	−3.106200	0.2132179	686	−14.568195	<0.0001
S8/C6	−1.175200	0.2132179	686	−5.511732	<0.0001
S8/C9	−4.920200	0.2132179	686	−23.075924	<0.0001
S12/C3	−5.342000	0.2132179	686	−25.054182	<0.0001
S12/C6	−4.087200	0.2132179	686	−19.169123	<0.0001
S12/C9	−5.547000	0.2132179	686	−26.015640	<0.0001
S16/C3	−5.956200	0.2132179	686	−27.934803	<0.0001
**S16/C6**	−5.101000	0.2132179	686	−23.923883	<0.0001
**S16/C9**	−5.961200	0.2132179	686	−27.958253	<0.0001
**S20/C3**	−6.392200	0.2132179	686	−29.979660	<0.0001
**S20/C6**	−5.820200	0.2132179	686	−27.296958	<0.0001

DF, Degree of freedom.

Table 7 summarises the findings of comparable retrospective studies performed to determine the palatal bone thickness and the most favourable sites for MI insertion, taking into consideration age and sex factors when applicable, in addition to the present findings.

**Table 7.** A summary of the conclusions of comparable retrospective studies, in addition to our results evaluating palatal bone thickness.

Author	Population	Measured sites	Results
Andraws Yalda et al. (2024) Iraqi study	68 Kurdish-Iraqi participants (37 M, 31 F; age range = 18–30 years)	The T-zone points at 3 mm from the MPS at the level of the palatal cusps of second premolars, and the posterior point at the level of mesio-palatal cusps of first molars	• Palatal bone is thinner in the posterior area compared to the anterior region • The T-zone is favourable for MI insertion. However, CBCT is recommended due to individual variability • Palatal bone thickness significantly decreases with age • Similar palatal bone thickness among male and female individuals • Similar paired parasagittal palatal bone thickness • Age influence on bone thickness was not examined
**Bonangi et al. (2018**) Indian study	30 individuals (age range = 12–28 years; number of each sex is not reported)	4, 8, 12, 16, 20 and 24 mm posterior to the IF and 0, 3 and 6 mm parasagittal to the MPS	• Greater bone thickness sites are the median and paramedian sites, 4 mm and 8 mm, posterior to the IF. Those sites are suitable for MI insertion • Palatal bone is thinner in the posterior area compared to the anterior region • Boys/men had a significantly thicker palatal bone compared to girls/women
**Chhatwani et al. (2019)** German study	180 participants (95 M, 85 F, age range = 8–40 years) ; divided into four age groups	88 points, 2 mm apart, from 0 mm to 14 mm posterior to IF and 10 mm lateral to the MPS	• Greater palatal bone thickness median and paramedian palatal regions, 4 mm posterior to the IF, which are suitable for MI insertion, followed by the area at 8 mm and least at 24 mm • Palatal bone is thinner in the posterior area compared to the anterior region • Boys/men had a significantly thicker palatal bone compared to girls/women • Palatal bone thickness significantly decreases with age
Chang et al. (2021) Taiwanese study	43 participants; group 1 (22 adults, mean age = 23.64 ± 4.18 years); group 2 (21 adolescents, mean age = 14.29 years)	Bone thickness was extracted from 32 paired points per patient, posterior to the IF with a 3-mm interval (paired point measurements were averaged to one value)	• Greater palatal bone thickness 3 mm distal to the IF and 4 and 8 mm lateral to the MPS which are favourable for MI placement • In adolescents, it is advisable to insert MI 6 mm posterior to the IF and 2 mm to 8 mm lateral to the MPS to avoid incisor root damage • Similar palatal bone thickness for both age groups in the paramedian posterior palate area • Palatal bone becomes thinner from anterior to posterior and from medial to lateral sides • No significant sex difference in palatal bone thickness
Gracco et al. (2008) Italian study	162 participants (80 M, 82 F; group A: age 10–15 years; group B: age 15–20 years; group C: 72 patients (aged 20–44 years)	At 4, 8, 16 and 24 mm posterior to the IF and 0, 3 and 6 mm lateral to the MPS	• The thickest part of the palate is the anterior region, but bone thickness in the posterior region is also suitable for screws of appropriate diameter and length • No significant sex difference in palatal bone thickness • No significant difference in bone thickness among the age groups at 4, 6 and 8 mm posterior to the IF except at 24 mm posterior to the IF and 6 mm lateral to the MPS • No significant difference in bone thickness at the MPS or at 3 and 6 mm lateral to the MPS, until point 24 mm posterior to the IF
Jayakumar et al. (2012) Japanese study	Skulls of individuals aged 18–65 years, with a mean age of 35.3 years	A reference plane and a plane parallel to this plane at the posterior margin of the IF were set. The measurements were performed at each of the six cross-sectional sites	• Palatal bone becomes thinner from anterior to posterior and from medial to lateral sides • The bone thickness was greater at the MPS • Age and sex confounders were not analysed
Nishi et al. (2014) Four ethnic groups	160 participants aged 16–45 years, divided into four equal ethnic groups (20 M, 20 F)	130 points between the incisive fossa to the palatal root of the maxillary first molar and 10 mm parasagittal of the MPS	• The palatal bone thickness is significantly greater in the paramedian sites in the anterior zone and the median area in the posterior zone. These sites are suitable for MI placement • Boys/men had a significantly thicker palatal bone compared to girls/women • A trend of decreasing thickness was as follows: Caucasian > Hispanic > African American > Asian
Wang et al. (2017) Chinese study	107 participants; 51 adolescents (16 M, 32 F) and 56 adults (25 M, 31 F)	72 palatal sites at 2, 4, 6 and 8 mm lateral to the MPS; 0–24 at 3 mm interval posterior to the IF	• Palatal bone becomes thinner from anterior to posterior and from medial to lateral sides • No significant sex difference in palatal bone thickness • No significant difference in bone thickness between paired parasagittal sites • No significant difference in bone thickness between adolescents and adults
Yadav et al. (2018) American study	359 participants; 204 adolescents (99 M, 105 F) and 155 adults (74 M, 81 F)	Palatal bone thickness 4 mm parasagittal between the canine and first premolar, first premolar and second premolar, second premolar and first molar, and first molar and second molar	• Greatest bone thickness in the anterior part of the palate, between canine and first premolar • The thickness tends to decrease progressively toward the posterior palatal sites • Boys/men had a significantly thicker palatal bone compared to girls/women • Palatal bone thickness significantly decreases with age
The present Portuguese study	50 participants; adolescents (13 M, 12 F; age range = 12–25 years) and 25 adults (10 M, 15 F; age range = 27–51 years)	Palatal bone thickness on 15 sites in the right side of each palate, 4, 8, 12, 16 and 20 mm distal to the IF and 3, 6 and 9 mm lateral to the midline	• Palatal bone thickness decreases from anterior to posterior direction • Anterior region of the palate is a highly favourable location for orthodontic MІ placement • Girls/women generally have a thinner palatal bone compared to boys/men • Palatal bone thickness tends to be greater in the adolescent and young adult age group

CBCT, cone-beam computed tomography; F, female; IF, incisive foramen; M, male; MI, micro implant; MPS, midpalatal suture.

## Discussion

### Summary

This study was a retrospective, 3D radiographic observational investigation conducted on a cohort of Portuguese patients attending Egas Moniz University Clinics. Similar to dental implants, the success of MІ is heavily influenced by the quantity and quality of the surrounding bone, particularly cortical bone density and thickness. These factors are vital for establishing primary stability, which is a pivotal element for ensuring long-term success (Chang et al., 2021; Vidalon et al., 2021). This study concluded that boys/men had significantly thicker palatal bone than girls/women. The palatal bone was significantly thicker in the adolescent and young adult age group compared to the adult age group. In addition, the bone thickness decreased posteriorly within each sagittal section.

### Generalisability

MI use is generally recommended when bone maturation is adequate to ensure retention and stability. This normally occurs around the age of 16 years in girls and 18 years in men (Chen et al., 2007). Markedly, no difference in palatal MI stability and retention was observed among adults, adolescents and even children, indicating that the palate bone density, even at an early age, is sufficient for MI stability along an orthodontic treatment (NienKemper et al., 2015; Wilmes et al., 2016). In our investigation, the sample size consists of patients aged 12–51 years. This age range was determined by several practical considerations, including the limited availability of CBCT scans and the necessity of fulfilling the inclusion criteria, such as full upper arch permanent dentition. This criterion is inherently age-dependent, as the prevalence of a complete dental arch tends to decrease with advanced age. Notably, this age range aligns with some previously undertaken studies, likely due to the necessity of meeting similar inclusion criteria (Chang et al., 2021; Chhatwani et al., 2019; Wilmes et al., 2016). However, it would be reasonable to narrow the age range by adding more groups with greater sample sizes in future studies.

The acquired CBCT images were oriented using landmarks on the palatal bone before taking measurements. This standardisation was focused at proximity to the region of interest (hard palate), a crucial step in ensuring uniformity across CBCT scans. A comparable standardised positioning was also employed in numerous similar studies (Bonangi et al., 2018; Santiago et al., 2020; Suteerapongpun et al.,2018). This meticulous approach is essential for minimising measurement variability and ensuring the reliability of subsequent analyses. On the other hand, Vidalon et al. (2021) used the Frankfurt horizontal plane as a guide for their image orientation. Comparable measurements are reported from the studies using different orientation landmarks, indicating that the accurate standardisation of the images is more important than the referenced landmarks.

The majority of the studies investigated the total palatal bone thickness with the main aim of guiding the insertion of palatal MІ and decreasing the failure rate in clinical practice. Our study further categorised the participants into age and sex groups to similarly investigate the effect of age and sex on palatal bone thickness. Andraws Yalda et al. (2024), Chang et al. (2021), Gracco et al. (2008) and Wang et al. (2017) concluded that no significant sex difference was observed in their participants. On the other hand, Bonangi et al. (2018), Chhatwani et al. (2019) and Yadav et al. (2018) reported a greater palatal bone thickness in boys/men than in girls/women, aligning with our findings. This discrepancy in the outcome might be due to the different age ranges in each group and the ethnicities reported in other studies. In addition, contrary to ours, most previous investigations did not perform a sample size power calculation. Another significant factor is that the vertical dimension was not considered in all the reviewed studies, including ours. Vidalon et al. (2021) noticed that participants with hypodivergent growth patterns had significantly greater palatal bone height and increased cortical thickness compared to those with hypodivergent and normodivergent growth forms. Investigating the impact of growth patterns on palatal bone thickness was not possible in our study because the field of view of the CBCT was confined to the protocol of As Low As Diagnostically Acceptable being Indication oriented and Patient Specific (ALADAІP) (Oenning et al., 2018). Therefore, obtaining CBCTs, including the total maxillomandibular structures, was unjustified.

Given the 15 anatomically defined points where palatal bone thickness was measured in this study, we addressed the issue of multiple comparisons using the BH procedure to control the FDR. This approach was selected over the more conservative Bonferroni correction to preserve statistical power while still maintaining adequate control of false positives (Korthauer et al., 2019; Verhoeven et al., 2005). The BH method is particularly appropriate in the context of anatomical or exploratory studies involving moderate numbers of comparisons, where the goal is to detect meaningful patterns without excessively inflating type II errors (Korthauer et al., 2019; Verhoeven et al., 2005). In our analysis, no clustering of borderline *P* values or excessive detection of significance was observed after BH adjustment, suggesting that the probability of spurious findings remained low. Furthermore, the measurement sites were not chosen arbitrarily but were anatomically grounded based on established insertion zones in the literature (Andraws Yalda et al., 2024; Bonangi et al., 2018; Chang et al., 2021; Chhatwani et al., 2019; Gracco et al., 2008; Jayakumar et al., 2012; Ludwig et al., 2011; Suteerapongpun et al., 2018; Vidalon et al., 2021; Wang et al., 2017; Yadav et al., 2018). Thus, the statistical inferences derived from these points are both clinically meaningful and methodologically sound. The use of FDR correction permitted us to maintain interpretative clarity while ensuring that our conclusions regarding sex- and age-related variations, and the identification of favourable insertion sites were not the result of random fluctuation.

The results of the present study revealed a significant decrease in palatal bone thickness from the anterior to the posterior regions. The highest mean vertical palatal bone thickness was observed in the anterior region of the hard palate, specifically at the S4C9 point, located 4 mm posterior to ІF and 9 mm laterally to MPS in all investigated groups aligning with all of our cited studies (Andraws Yalda et al., 2024; Bonangi et al., 2018; Chang et al., 2021; Chhatwani et al., 2019; Jayakumar et al., 2012; Ludwig et al., 2011; Negrisoli et al., 2022; Suteerapongpun et al., 2018; Vidalon et al., 2021; Wang et al., 2017; Yadav et al., 2018). Although the smallest mean vertical palatal bone height was located in the distal region of the hard palate, 20 mm posterior from the ІF and 6 mm lateral to the MPS. This result was comparable with those of other studies where the thinnest palatal bone was observed within 20–24 mm posterior to the ІF (Andraws Yalda et al., 2024; Bonangi et al., 2018; Chang et al., 2021; Chhatwani et al., 2019; Gracco et al., 2008; Jayakumar et al., 2012; Ludwig et al., 2011; Suteerapongpun et al., 2018; Vidalon et al., 2021; Wang et al., 2017; Yadav et al., 2018). Therefore, the first null hypothesis was rejected. Insertion of the MІ in the anterior segment of the palate presents significant advantages. The favourable anatomical conditions, including the relative absence of vital anatomical structures, adequate bone volume and sufficient keratinised gingiva, suggest a definitely lower failure rate for MІ insertion compared to inter-root insertion (Іodice et al., 2022).

Similar to Bonangi et al. (2018), Chhatwani et al. (2019), Negrisoli et al. (2022) and Yadav et al. (2018), our investigation revealed that boys/men exhibited significantly greater palatal bone thickness than girls/women, leading to the rejection of the second null hypothesis. This finding has a high clinical relevance for determining the acceptable MI length used in the palate. The sex-specific pattern has been reported for other areas of the jaws, like the alveolar bone, where females seem to have thinner bone compared to boys/men (Firincioglulari et al., 2024). In contrast, some studies revealed that girls/women have an equal or greater bone supply in the posterior region of the palate than boys/men (Chang et al., 2021; Gracco et al., 2008; Sumer et al., 2016). A possible explanation for these discrepancies is the use of disparate measurement techniques and protocols or differences in the composition of the study populations, including potential ethnic variations.

The present study reveals that adolescents and young adults exhibited greater average palatal bone thickness compared to adults, thus rejecting the third null hypothesis. The same results were obtained by Chhatwani et al. (2019) in 180 male and female healthy German orthodontic patients aged 8–40 years. Yadav et al. (2018) reported similar findings among 359 American participants. Santiago et al. (2020) also revealed that the palatal bone thickness has a significant but weak correlation with the age of their examined cohort. On the contrary, other comparable investigations found no age difference in palatal bone thickness (Chang et al., 2021; Gracco et al., 2008; Wang et al., 2017). A potential reason for these outcome discrepancies is the variations in the investigated cohorts and the different population ethnicities.

### Interpretation

Palatal bone thickness measurements are instrumental in selecting optimal MІ placement sites and determining appropriate implant length. This information is critical for achieving sufficient implant stability and preventing iatrogenic injury to vital anatomic structures. It was suggested that a minimum of 5 mm of bony support is crucial for resisting rotational forces and dynamic loads, thereby contributing to MІ stability, and preventing iatrogenic injury to vital anatomical structures (Winsauer et al., 2014). Ludwig et al. (2011) reported that the ideal area for MІ insertion is 3–9 mm posterior to the ІF and within 6 mm mediolaterally including the MPS.

The results of the cluster analysis indicated two groups of sites with a high degree of homogeneity. Integrating the CBCT-driven cluster analysis results into clinical workflows enables more precise, patient-specific treatment planning, reducing risks and improving long-term outcomes in implantology, orthodontics and maxillofacial surgery. Accordingly, the favourable MI insertion sites in our cohort are located 4–8 mm posterior to the ІF and within 9 mm mediolaterally for both male and female groups. Regarding age, the preferred insertion site is located 4–8 mm posterior to the ІF and 3–9 mm lateral to the MPS for adults and 4–12 mm posterior to the ІF and 3–9 mm lateral to the MPS for adolescents and young adults. The results of the present study also confirm previous findings that the T-zone, the area just posterior to the third palatal rugae in the anterior region of the palate, is one of the most favourable sites in the hard palate for MІ insertion (Hourfar et al., 2015; Ludwig et al., 2011; Wilmes et al., 2016). These findings enhance our knowledge regarding palatal bone and provide clinical guidelines that could reduce the necessity for radiographic imaging before the insertion of the orthodontic MІ. However, further research, based on a multicentre cohort of participants, would improve generalisability.

Despite the reported findings, careful diagnostic evaluation remains essential for each patient. Assessment of palatal bone thickness is critical in orthodontic practice due to its implications for MІ placement. Thicker bone regions offer enhanced MІ stability, while thinner areas increase the risk of MІ failure and iatrogenic injury. Individual variations in bone thickness, influenced by sex and age, necessitate personalised treatment plans. Accurate assessment guides optimal MІ placement site and length, maximising success rates and minimising complications. This clinical relevance underscores the need for thorough bone thickness evaluation before MІ insertion and further research in this area to refine treatment protocols and improve patient outcomes.

The ongoing advancements in artificial intelligence (AІ) and the emerging field of computer graphics are poised to revolutionise landmark evaluation. The integration of these technologies is expected to facilitate self-automated assessment processes that are both efficient and reliable (Radwan et al., 2023). These AІ-powered systems can accurately identify and segment palatal bone, enabling precise measurements of bone thickness at various locations. This automated approach not only streamlines the assessment process but also enhances its accuracy and reliability, ultimately leading to better-informed treatment decisions and improved patient outcomes.

### Limitations

Despite the relevant outcome of the present investigation, several limitations should be addressed in future similar research. First, the retrospective design limited control over the imaging procedures, which might have introduced bias by excluding patients with incomplete clinical data or distorted CBCT scans. Second, although the achieved power of the sample size (n = 50) was notably high, suggesting a robust statistical foundation, this cohort was not matched by sex and age. In addition, the cohort represents individuals attending a single university clinic. Future research should employ a prospective multicentre design that includes participants matched for sex and age from various dental clinics across Portugal, enhancing the generalisability of the findings. Therefore, this study should be considered as a foundational step for future investigations. Furthermore, despite the rigorous training and the high accuracy of the intra-examiner analysis, an inter-examiner evaluation conducted by an experienced researcher in the field is recommended in future studies.

## Conclusions

This study investigated the suitability of the palatal bone for MІ placement by analysing variations in palatal bone thickness based on anatomical location, age and sex. Our findings reveal the following key points:

- decreasing anteroposterior thickness: palatal bone thickness decreases from the anterior to posterior direction.
- favourable insertion sites: anterior region of the palate is a highly favourable location for orthodontic MІ placement due to its sufficient bone thickness, contributing to optimal primary stability.
- sex differences: girls/women generally have a thinner palatal bone compared to boys/men.
- age-related differences: palatal bone thickness tends to be greater in the adolescents and young adults age group (aged 12–25 years) than in adults (aged 27–51 years).
